# Testosterone and Voluntary Exercise, Alone or Together Increase Cardiac Activation of AKT and ERK1/2 in Diabetic Rats

**DOI:** 10.5935/abc.20160174

**Published:** 2016-12

**Authors:** Leila Chodari, Mustafa Mohammadi, Gisou Mohaddes, Mohammad Reza Alipour, Vajiheh Ghorbanzade, Hassan Dariushnejad, Shima Mohammadi

**Affiliations:** 1 Drug Applied Research Center, Tabriz University of Medical Sciences; Tabriz - Irã; 2 Neuroscience Research Center of Tabriz University of Medical Sciences, Tabriz - Irã

**Keywords:** Testosterone, Rats, Diabetes Mellitus: Angiogenesis, Exercise, Proto-Oncogenese Proteins C-akt, Extracellular Signal-Regulated MAP Kinases

## Abstract

**Background:**

Impaired angiogenesis in cardiac tissue is a major complication of diabetes. Protein kinase B (AKT) and extracellular signal regulated kinase (ERK) signaling pathways play important role during capillary-like network formation in angiogenesis process.

**Objectives:**

To determine the effects of testosterone and voluntary exercise on levels of vascularity, phosphorylated Akt (P- AKT) and phosphorylated ERK (P-ERK) in heart tissue of diabetic and castrated diabetic rats.

**Methods:**

Type I diabetes was induced by i.p injection of 50 mg/kg of streptozotocin in animals. After 42 days of treatment with testosterone (2mg/kg/day) or voluntary exercise alone or in combination, heart tissue samples were collected and used for histological evaluation and determination of P-AKT and P-ERK levels by ELISA method.

**Results:**

Our results showed that either testosterone or exercise increased capillarity, P-AKT, and P-ERK levels in the heart of diabetic rats. Treatment of diabetic rats with testosterone and exercise had a synergistic effect on capillarity, P-AKT, and P-ERK levels in heart. Furthermore, in the castrated diabetes group, capillarity, P-AKT, and P-ERK levels significantly decreased in the heart, whereas either testosterone treatment or exercise training reversed these effects. Also, simultaneous treatment of castrated diabetic rats with testosterone and exercise had an additive effect on P-AKT and P-ERK levels.

**Conclusion:**

Our findings suggest that testosterone and exercise alone or together can increase angiogenesis in the heart of diabetic and castrated diabetic rats. The proangiogenesis effects of testosterone and exercise are associated with the enhanced activation of AKT and ERK1/2 in heart tissue.

## Introduction

Diabetes mellitus is a metabolic disease with a high and increasing prevalence worldwide.^1^ Cardiovascular complications are the main causes of mortality and morbidity in diabetic subjects and suppression of angiogenesis in coronary heart and peripheral vascular system can possess influential effects in this case.^2^ AKT and ERK are two major signaling pathways that play central role in regulating cell proliferation, migration, and survival through their downstream targets.^3,4^ There are evidences that AKT-dependent signaling pathways have a critical role in the regulation of cardiac growth, contractile function, and coronary angiogenesis.^5,6^ It has been proved that AKT signaling pathway enhances angiogenesis in the heart tissue.^7^ MAPK-ERK signaling plays an important role during angiogenesis and is necessary for endothelial cell sprouting.^8^

Testosterone is the major gonadal androgen in men. Testosterone deficiency is common in men with diabetes and male STZ-induced diabetic rats.^9^ Also, it is shown that cardiovascular disease increase with testosterone deficiency.^10^ These observations may indicate that testosterone supplementation in diabetic men would be beneficial in attenuating cardiovascular disease.

Exercise has beneficial effects on functional properties of both healthy and diabetic heart muscle. Also, exercise training improves blood glucose metabolism, insulin action and cardiovascular risk factors in diabetic subjects.^11^ Voluntary exercise as a mild/moderate exercise^12^ have been shown to decrease cardiovascular disease.^11^ In the animal model of voluntary exercise, the animal has free access to a running wheel and uses the wheel according its physiological threshold for physical activity^12^.

Although the cardiovascular system is an important target of exercise and androgen action, the molecular mechanism of action of testosterone and voluntary exercise in heart of diabetics remains largely unexplored. So, the aim of this study was to investigate the effect of testosterone replacement therapy and exercise training, alone or in combination on P-Akt and P-ERK levels (as proangiogenic factors) in the heart tissue of diabetic and castrated diabetic rats.

## Methods

### Animals

Animals used in this study were provided by the colony of our university. The animals were housed in a temperature-controlled facility (21 -23°C), maintained on a 12:12-h light-dark cycle with food and water provided ad libitum. All animal experiments were performed under the guidelines on human use and care of laboratory animals for biomedical research published by National Institutes of Health (8th ed., revised 2011) and conformed to the Declaration of Helsinki. The Ethics Committee of Tabriz University approved the experimental protocol. Sixty-three male Wistar rats (250 - 270 g) were randomly assigned to testosterone or placebo treatment and sedentary or voluntary exercise groups. Then, the animals were divided into nine groups (n= 7): 1- Diabetic sham castration + placebo group (Dia-S- Cas), 2-Diabetic + placebo group (Dia), 3-Diabetic + Testosterone group (Dia -T), 4-Diabetic + Exercise + placebo group (Dia-E), 5-Diabetic + Exercise + Testosterone group (Dia-T-E), 6-Diabetic + castrated + placebo group (Dia-Cas), 7- Diabetic + castrated + Testosterone group (Dia-Cas-T), 8-Diabetic + castrated + Exercise + placebo group (Dia-Cas-E) ,9-Diabetic + castrated + Testosterone+ Exercise group (Dia-Cas-T-E) .

### Castration and hormone replacement therapy

Sexually adult male rats were anesthetized with ketamine hydrochloride (80 mg/kg) and xylazine hydrochloride (5 mg/kg). Then, animals were placed in a supine position and their testes were removed via a low-middle abdominal incision or remained intact. To avoid disruption of hormonal influence, testosterone replacement began immediately after surgery.^13^ Testosterone propionate (UNIGEN, Life Science) dissolved in dimethyl sulfoxide (DMSO) and administered subcutaneously at a physiological dose (2 mg/kg /day) for 6 weeks.^14^ Rats in the Dia-S- Cas, Dia, Dia-E, Dia-Cas and Dia-Cas-E groups were injected with the same amount of DMSO vehicle.

### Induction of diabetes

All animals were injected with streptozotocin (50 mg/kg) (Sigma, St. Louis Mo, USA), to induce type I diabetes.^15^ Streptozotocin was dissolved in 10 mM sodium citrate, pH 4.5, with 0.9% NaCl. Diabetes was verified 72 h later by evaluating blood glucose levels by using glucometer (Elegance, Model: no: CT-X10 Germany). Rats with a blood glucose level ≥300 mg/dL were considered to be diabetic.^16^ Induction of diabetes in animals was performed seven days after castration surgery.

### Voluntary exercise

Voluntary exercise is considered as mild / moderate exercise. In this study, rats in exercise groups were housed individually in cages that were equipped with a stainless-steel vertical running wheel (Tajhiz Gostar, Tehran) and were allowed free access to the wheel 24 h per day for 6 weeks. The wheels were bound to a permanent sensor that activated a digital counter of wheel revolutions. Total wheel circulations were recorded daily, with total distance run per day determined by multiplying the number of wheel rotations by wheel circumference. Animals that had run less than 2000 m/day were replaced.^15^

### Tissue processing and protein measurement

At the end of study, heart tissues were excised and frozen in liquid nitrogen immediately. Tissue samples from left ventricle were stored in -70 °C temperature until phospho-AKT (serine 473) and phospho-ERK 1/2 (threonine 202/tyrosine204) measurements. In order to measure protein levels in heart tissue, samples were homogenized in PBS (pH 7.2-7.4) and centrifuged for 20 min at the speed of 3000 rpm at 4°C. Resulting supernatants were removed and target proteins were extracted. Phosphorylated and activated forms of AKT (Eastbiopharm Co., Ltd., Hangzhou, China) and ERK1/2 (Abcam, Cambridge, U.K.) levels were measured by high-sensitivity ELISA and normalized to the total protein concentration for each sample as determined by the Bradfored assay.

### Histological evaluation

Tissue samples from left ventricle were immediately isolated and fixed in 10% buffered-formalin solution, dehydrated in ascending grades of alcohol and embedded in paraffin. Sections of 5 µm were taken, stained with hematoxylin-eosin (H-E), and examined under light microscope (Olympus BH-2, Tokyo, Japan) in a blinded manner. Heart tissues were evaluated in terms of interstitial edema, congestion, leukocytosis infiltration, and myonecrosis.

### Angiogenesis assay

Angiogenesis was measured by microvasculature density (MVD) in five sections at 50mm intervals from the intensely vascularized area under a light microscope by two independent observers who were blinded to the experimental conditions. The numbers of microvessels from each section were counted and calculated for the average number of microvessels per section.^17^

### Statistical analysis

Data are expressed as means± SEM. After having evaluated the homogeneity of variance and normal distribution of data, one or two-way analysis of variance (ANOVA) and also Tukey's test was used to compare quantitative data. Mann-Whitney U test was used to test histological variables. Statistical significance was defined as p<0.05. Statistical analysis of data was carried out using SPSS statistical software (Version 17.0).

## Results

### Effects of testosterone and voluntary exercise on P- AKT levels in the heart tissue of type I diabetic and diabetic castrated rats

**Figure 1:**
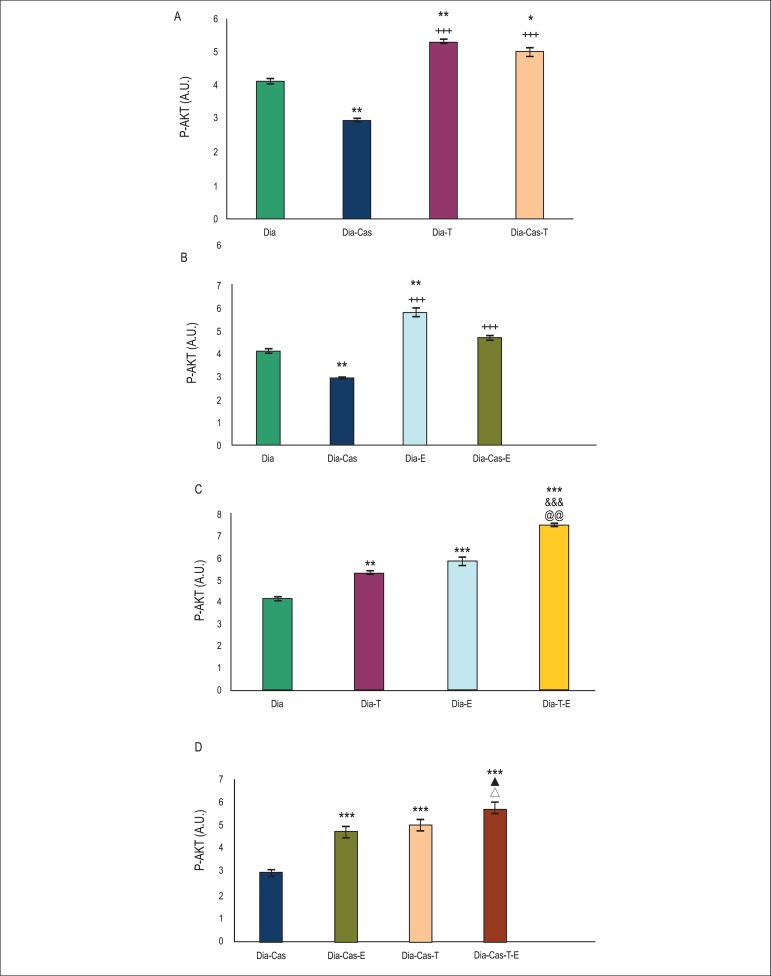
A) Effect of 6 weeks testosterone treatment on P-AKT protein levels in the heart tissue of diabetic and diabetic castrated groups; B) Effect of 6 weeks exercise training on P-AKT protein levels in the heart tissue of diabetic and diabetic castrated groups; C) Effects of 6 weeks testosterone and exercise on P-AKT protein levels in the heart tissue of the diabetic group; D) Effects of 6 weeks testosterone and exercise on P-AKT protein levels in the heart tissue of the diabetic castrated group. Data are expressed as mean± SEM for 7 animals. * p<0.05, ** p<0.01, *** p<0.001 vs the Dia group, &&& p<0.001 vs the Dia-T group, @@ p<0.01 vs the Dia-E group, +++ p<0.001 vs the Dia-Cas group, ▲ p<0.05 vs the Dia-Cas-E group, Δ p<0.05vs the Dia-Cas-T group.

One-way ANOVA showed that castration significantly (p<0.01) decreased P- AKT protein levels in the heart tissue of diabetic rats. Treatment of diabetic (p<0.01) and diabetic castrated (p<0.05) groups with testosterone significantly raised P-AKT protein levels in the heart tissue compared to diabetes group (Figura 1A). Figure 1 B (analysis by one way ANOVA) shows that six weeks treatment of diabetic (p<0.01) and diabetic castrated (p<0.001) groups with exercise significantly raised P-AKT protein levels in heart compared to diabetic and diabetic castrated groups, respectively. Two way ANOVA in figure 1C shows that combination therapy with testosterone and exercise in diabetic rats significantly (p<0.001) enhanced P-AKT protein levels in the heart tissue in comparison with diabetes group. Also, combination therapy with testosterone and exercise in diabetic rats showed a significant increasing effect on P-AKT protein levels compared to testosterone (p<0.001) or exercise (p<0.01) groups.

Figure 1D shows that in diabetes-castration group, six weeks combination therapy with testosterone and exercise significantly (p<0.001) elevates P-AKT protein levels in the heart tissue in comparison with diabetes castrated group. Also, combination therapy with testosterone and exercise in castrated diabetic rats showed a significant (p<0.05) increasing effect on P-AKT protein levels compared to testosterone or exercise groups. Analysis of data for sham group showed no significant difference in comparison to diabetic group (data are not shown).

### Effects of testosterone and voluntary exercise on P-ERK 1/2 protein levels in the heart tissue of type I diabetic and diabetic castrated rats

**Figure 2:**
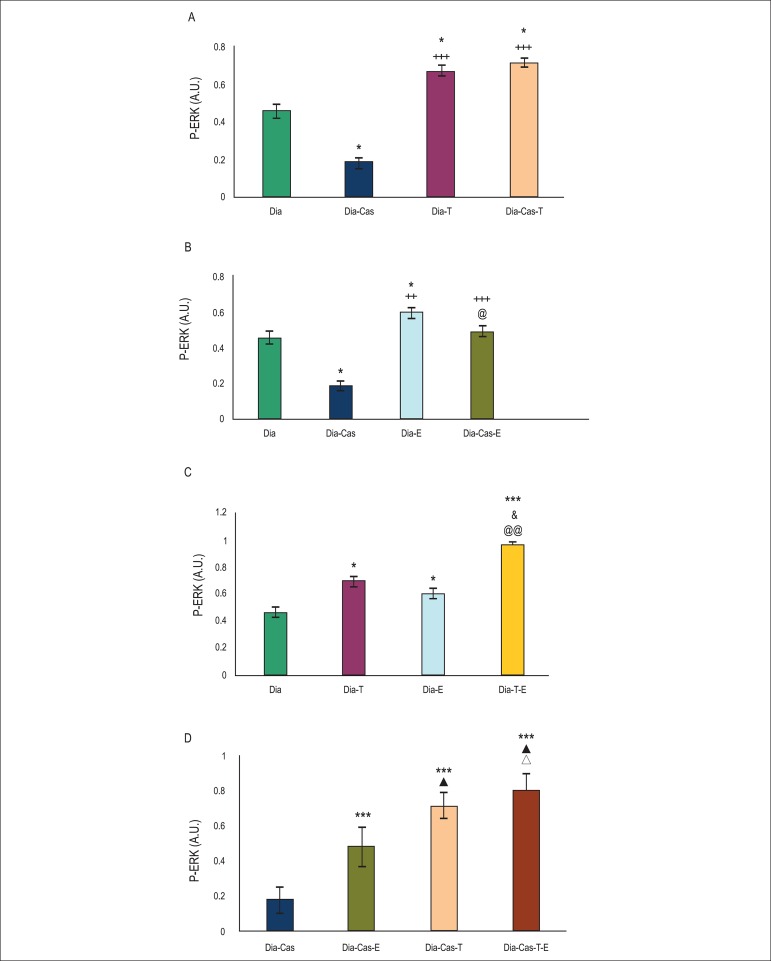
A) Effect of 6 weeks testosterone treatment on P-ERK 1/2 protein levels in the heart tissue of diabetic and diabetic castrated groups; B) Effect of 6 weeks exercise training on P-ERK 1/2 protein levels in the heart tissue of diabetic and diabetic castrated groups; C) Effects of 6 weeks testosterone and exercise on P-ERK 1/2 protein levels in the heart tissue of diabetic group; D) Effects of 6 weeks testosterone and exercise on P-ERK 1/2 protein levels in the heart tissue of the diabetic castrated group. Data are expressed as mean± SEM for 7 animals. *p<0.05, *** p<0.001 vs the Dia group. & p<0.05 vs the Dia-T group. @ p<0.05, @@ p<0.01 vs the Dia-E group. + p<0.05, ++ p<0.01 , +++ p<0.001 vs the Dia-Cas group. ▲ p<0.05 vs the Dia-Cas-E group. Δ p<0.05 vs the Dia-Cas-T group.

One-way ANOVA showed that in the diabetes-castration group, P-ERK 1/2 protein levels in the heart tissue decreased significantly (p<0.05) in comparison to the diabetic group. Six weeks treatment of diabetic (p<0.05) and diabetic castrated (p<0.001) groups with testosterone significantly enhanced P-ERK 1/2 protein levels in the heart tissue in comparison to Dia and Dia-Cas groups, respectively (Figure 2A).

Figure 2B (analysis by one way ANOVA) shows that six weeks treatment of diabetic (p<0.05) and diabetic castrated (p<0.001) groups with exercise significantly raised P-ERK 1/2 protein levels in the heart compared to diabetic and diabetic castrated groups, respectively.

According to figure 2C (analysis by two way ANOVA), six weeks of combination therapy with testosterone and exercise in diabetic rats significantly (p<0.001) enhanced P-ERK 1/2 protein levels in the heart tissue in comparison with diabetes group. Also, combination therapy with testosterone and exercise in diabetic rats showed a significant increasing effect on P-ERK 1/2 protein levels compared to testosterone (p<0.05) or exercise (p<0.01) groups.

Figure 2D shows that in the diabetes-castration group, six weeks of combination therapy with testosterone and exercise significantly (p<0.001) increased P-ERK 1/2 protein levels in the heart tissue in comparison with the diabetes castrated group. Also, combination therapy with testosterone and exercise in castrated diabetic rats showed a significant (p<0.05) increasing effect on P-ERK 1/2 protein levels compared to testosterone or exercise groups. Analysis of data for the sham group showed no significant difference in comparison to diabetic group (data are not shown).

### Histopathological findings

#### Effects of testosterone and voluntary exercise on myocardial tissues in type I diabetic and diabetic castrated rats

**Figure 3:**
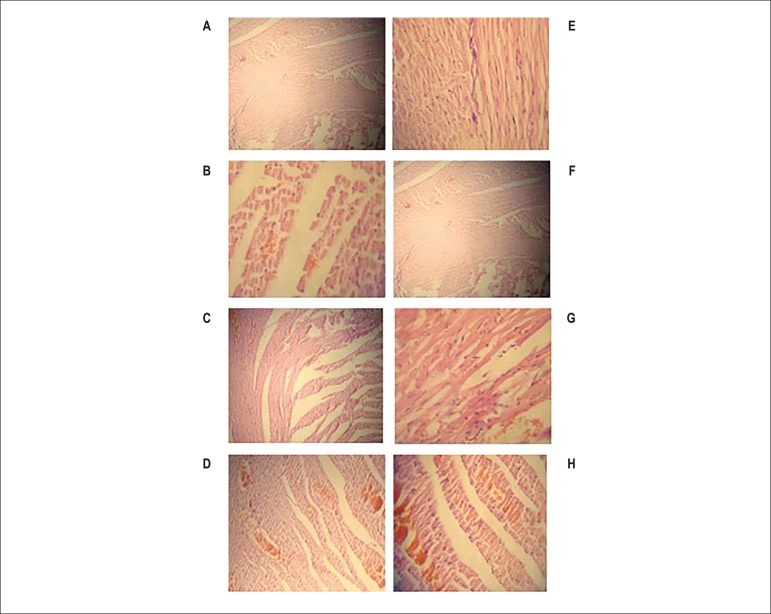
A-H) The histological light microscopy images of the left ventricle tissues stained with hematoxylin and eosin (40 × HE). A) Dia group; B) Dia-T group; C) Dia-E group; D) Dia-T-E group; E) Dia- Cas group; F) Dia- Cas-T group; G) Dia- Cas-E group; H) Dia- Cas-T-E group.

According to figure 3 and table 1A that analyzed by Mann-Whitney U test, castration increased interstitial edema (significantly p<0.05), leukocyte infiltration, and myonecrosis in the diabetes group. In the diabetic group, testosterone treatment resulted in a marked attenuation of interstitial edema, leukocyte infiltration and myonecrosis (significantly p<0.05) in comparison to the diabetic group. Also, six weeks exercise trained rats in the diabetes group had decreased interstitial edema, leukocyte infiltration (significantly p<0.05) and myonecrosis (significantly p<0.001) (Table 1B). Combination therapy with testosterone and exercise in diabetic rats showed a decreasing effect on myocardial tissues damage in comparison to Dia, Dia-E and Dia-T groups (Table 1C). Table 1A showed that in the Dia-Cas group testosterone treatment resulted in a marked attenuation of interstitial edema (significantly p<0.05), leukocyte infiltration (significantly p<0.01) and myonecrosis (significantly p<0.001) in comparison to the Dia-Cas group. Also, six weeks exercise trained rats in Dia-Cas group had decreased interstitial edema (significantly p<0.01), leukocyte infiltration (significantly p<0.05) and myonecrosis (significantly p<0.01) in comparison to the Dia-Cas group (Table 1B). Mann-Whitney U analysis showed that combination therapy with testosterone and exercise in castrated diabetic rats decreased interstitial edema (significantly p<0.01), leukocyte infiltration and myonecrosis (significantly p<0.001) in comparison to the Dia-Cas group (Table 1D).

**Table 1:** A) Effect of 6 weeks testosterone treatment on histological changes of cardiomyocytes in the diabetic and diabetic castrated groups; B) Effect of 6 weeks exercise training on histological changes of cardiomyocytes in the diabetic and diabetic castrated groups; C) Effects of 6 weeks testosterone and exercise on histological changes of cardiomyocytes in the diabetic group; D) Effects of 6 weeks testosterone and exercise on histological changes of cardiomyocytes in the diabetic castrated group

**A**	**Interstitial edema**	**Leukocyte infiltration**	**Cardiomyocyte necrosis and congestion**
Dia	1.57 ± 0.78	2.42 ± 0.78	2.57 ± 0.53
Dia-Cas	2.71 ± 0.48*	2.8571 ± 0.37	3.0 ± 0
Dia-T	1.71 ± 0.75++	1 ± 1 ++	0.85 ± 0.89*+++
Dia-Cas-T	1.85 ± 0.69+	1.42 ± 0.78* ++	1.42 ± 0.05**+++
			
**B**	**Interstitial edema**	**Leukocyte infiltration**	**Cardiomyocyte necrosis and congestion**
Dia	1.57 ± 0.78	2.42 ± 0.78	2.57 ± 0.53
Dia-Cas	2.71 ± 0.48*	2.8571 ± 0.37	3.0 ± 0
Dia-E	1.42 ± 0.53	1.28 ± 1*	1.0 ± 0***
Dia-Cas-E	1.85 ± 0.37 ++	2.14 ± 0.69007+@	2.14 ±0.69++@@
			
**C**	**Interstitial edema**	**Leukocyte infiltration**	**Cardiomyocyte necrosis and congestion**
Dia	1.57 ± 0.78	2.42 ± 0.78	2.57 ± 0.53
Dia-T	1.71 ± 0.75	1.85 ± 0.37	0.85 ± 0.89*
Dia-E	1.42 ± 0.53	1.28 ± 1. 00 *	1.0 ± 0***
Dia-T-E	1.85 ± 0.37	2.14 ± 0.69*	2.14 ±0.69**
			
**D**	**Interstitial edema**	**Leukocyte infiltration**	**Cardiomyocyte necrosis and congestion**
Dia-Cas	2.71 ± 0.48	2.8571 ± 0.37	3.0 ± 0
Dia-Cas-T	1.85 ± 0.69+	1.42 ± 0.78++	1.42 ± 0.05+++
Dia-Cas-E	1.85 ± 0.37++	2.14 ± 0.69007+	2.14 ±0.69++
Dia-Cas-T-E	1.28 ± 0.48++▲	1.28 ± 0.48+++▲	1.28 ± 0.48+++▲

Data are expressed as mean± SEM for 7 animals.

* p<0.05,

** p<0.01,

*** p<0.001 vs the Dia group.

@ p<0. 05,

@@ p<0. 01 vs the Dia-E group.

+ p<0.05,

++ p<0.01,

+++ p<0.001 vs the Dia-Cas group.

▲ p<0.05 vs the Dia-Cas-E group.

#### Effects of testosterone and voluntary exercise on microvascular density (MVD) in type I diabetic and diabetic castrated rats

One-way ANOVA showed that castration significantly (p<0.01) decreased the number of MVD in the heart tissue of diabetic rats. Treatment of Dia and Dia-Cas groups with testosterone significantly (p<0.01) enhanced the number of MVD compared to Dia and Dia-Cas groups, respectively (Table 2A). Table 2B showed that in Dia and Dia-Cas groups six weeks exercise training (p<0.01) significantly increased the number of MVD in the heart tissue compared to Dia and Dia-Cas groups, respectively. According to table 2C (analysis by two way ANOVA), six weeks of combination therapy with testosterone and exercise in diabetic rats significantly (p<0.001) enhanced the number of MVD in the heart tissue in comparison with the diabetes group. Also, combination therapy with testosterone and exercise in diabetic rats showed a significant (p<0.01) increasing effect on the number of MVD compared to testosterone or exercise treated groups. Figure 2D (analysis by two way ANOVA), shows that in the diabetes-castration group, six weeks combination therapy with testosterone and exercise significantly (p<0.001) increased the number of MVD in the heart tissue in comparison with the diabetes castrated group. Also, combination therapy with testosterone and exercise in castrated diabetic rats showed a significant (p<0.05) increasing effect on the number of MVD compared to exercise treated groups. Analysis of data for the sham group showed no significant difference in comparison to diabetic group (data are not shown).

**Table 2:** A) Effect of 6 weeks testosterone treatment on MVD cardiac tissue of the diabetic and diabetic castrated groups; B) Effect of 6 weeks exercise training on MVD cardiac tissue of the diabetic and diabetic castrated groups; C) Effects of 6 weeks testosterone and exercise on MVD cardiac tissue of the diabetic group; D) Effects of 6 weeks testosterone and exercise on MVD cardiac tissue of the diabetic castrated

**A**	**Dia**	**Dia-Cas**	**Dia-T**	**Dia-Cas-T**
Microvascular density			**	
		**	++	++
	239.57±11.76	196.00±14.70	281.00±9.50	245.42±9.66
				
**B**	**Dia**	**Dia-Cas**	**Dia-E**	**Dia-Cas-E**
Microvascular density			**	
		**	++	++
	239.57±11.76	196.00±14.70	269.1429±12.42	226.57±28.17
				
**C**	**Dia**	**Dia-T**	**Dia-E**	**Dia-T-E**
Microvascular density				***
				&&
				@@
		**	**	
	239.57±11.76	281.00±9.50	269.1429±12.42	305.7143±9.4
				
**D**	**Dia-Cas**	**Dia-Cas-T**	**Dia-Cas-E**	**Dia-Cas-T-E**
Microvascular density				+++
		+++	+	▲
	196.00±14.70	245.42±9.66	226.57±28.17	259.14±7.96

Group Data are expressed as mean± SEM for 7 animals.

** p<0.01,

*** p<0.001 vs the Dia group.

&& p<0. 01vs the Dia-T group.

@@ p<0. 01 vs the Dia-E group.

+ p<0.05,

++ p<0.01,

+++ p<0.001 vs the Dia-Cas group.

▲ p<0.05 vs the Dia-Cas-E group.

## Discussion

In this study, we provided novel information that testosterone and exercise increased P-AKT and P-ERK 1/2 levels in the heart tissue of type I diabetic rats. Our findings showed that castration impaired the activation of AKT and ERK 1/2 in the heart tissue of diabetic rats and testosterone-replacement therapy increased levels of these proteins. Specifically, we demonstrated that simultaneous treatment of diabetic and castrated diabetic rats with testosterone and exercise had additive effect on activation of these proteins. The present study revealed that levels of P-AKT and P-ERK 1/2 enhanced in exercise-trained groups compared with sedentary groups. Also, testosterone and exercise induced histological changes such as tissue organization and vascularization in the heart tissue. Briefly, the damage in the myocardium of diabetic and castrated diabetic rats decreased with testosterone treatment or exercise training. Moreover, simultaneous treatment with testosterone and exercise increased vascularization in the heart tissue of diabetic and castrated diabetic rats.

The diabetic condition changes neovascularization mechanisms and impairs vascular homeostasis. As, angiogenesis and capillary density decreases in heart of diabetic rats.^18^ Similarly, clinical studies have shown an abnormality in endothelium dependent vasodilation in patients with diabetes.^19^ Diabetes has been shown to cause structural and functional disturbances in the myocardium through myocardial fibrosis, collagen formation, myocyte hypertrophy, mitochondrial dysfunction, and ROS accumulation.^20^ It has been found that cardiac expression of VEGF-A and its receptors decreased in diabetic rats and in the human myocardium with diabetes.^21^

Several factors can affect angiogenesis in the heart of diabetic subjects; testosterone and exercise are the most notable of these factors.^14,22^ As, androgens, including testosterone, mediate biological effects on all aspects of cellular mechanisms, including proliferation, differentiation, and homeostasis. Moreover, it is shown that testosterone involves in endothelial cell proliferation and angiogenesis as testosterone supplementation increases angiogenesis in the heart of castrated rats.^23^ Also, previous studies have shown that testosterone deficiency is common in diabetic men and STZ-induced diabetic rats.^24,25^ Clinical trials have reported a higher prevalence of hypogonadism in diabetic men when compared with non-diabetics.^24^ It is shown that type I diabetic subjects have lower free testosterone compared with control subjects.^26^ These observations and other scientific evidence suggesting that testosterone deficiency contributes or at least is associated with cardiovascular risk factors in diabetic subjects.^27,28^ In our study, testosterone therapy increased vascularity in the myocardium of Dia and Dia-Cas groups.

Since regular moderate-intensity physical activity can reduce the risk of cardiovascular disease, it is recommended for patients with type I diabetes (T1DM).^29^ Multiple lines of evidence suggest that exercise increases angiogenesis.^30^ Our results showed that exercise increased MVD and decreased interstitial, leukocyte infiltration, and myonecrosis in the heart tissue of Dia and Dia-Cas groups.

To elucidate the mechanisms of testosterone and exercise for promotion of angiogenesis, we assessed AKT and ERK1/2 in the angiogenesis-related proteins. PI3K/Akt and MEK/ERK are two major cellular signaling pathways, which are activated ^32^ and play a principal role during angiogenesis.^33^ AKT is expressed in heart tissue at high levels.^34^ It has been shown that PI3K-AKT and MAPK-ERK signaling axis regulates multiple critical steps in angiogenesis.^35,36^ Dobrzynski et al^37^ showed that STZ-diabetic rats had a significant reductions in cardiac AKT activity and insulin treatment of streptozotocin (STZ)-diabetic rats enhanced retinal AKT phosphorylation. Also, it has been reported that insulin pretreatment of hepatoma cells specifically enhanced growth hormone induced ERK1/2 phosphorylation.^33^

It is clear that testosterone increased both AKT and ERK1/2 phosphorylation in neonatal rat cardiomyocytes.^38^ It seems that testosterone increases the HIF-1a and VEGF-A levels^14^ which act as chief regulators of neovascularization through phospho-AKT/AKT pathway. Also, testosterone can enhance SDF-1a levels which decreases apoptosis and increases angiogenesis.^14^

Multiple lines of evidence suggest that exercise can increase angiogenesis through increasing VEGF and eNOS phosphorylation by AKT in the post-ischemic failing heart in rats.^39,40^ Also, it is revealed that exercise can activate ERK in rodent skeletal muscle.^41^ In this regard, we showed that voluntary exercise increases cardiac P-AKT and P-ERK in diabetic and castrated diabetic rats, which can lead to angiogenesis. Other possible mechanisms are increasing of VEGF-A and decreasing of oxidative stress by voluntary exercise.^16^

Furthermore, for the first time, our study showed that testosterone treatment and exercise training have an additive effect on diminution of tissue injury, vascularity and activation of AKT and ERK in the hearts of diabetic rats. In agreement with our study, Fry and Lohnes showed that resistance exercise can elicit a heightening testosterone response that partially explains the muscle hypertrophy observed in athletes.^42^ It is also reported that, when exercise is added to testosterone supplementation, cardiac remodeling process, as structural or biochemical changes, becomes more efficient.^43^ Regarding the limitations of this study and results of previous studies, we did not measure circulating testosterone after castration.

## Conclusion

Based on our findings, we conclude that testosterone and exercise increase angiogenesis and diminish histological abnormalities in the hearts of type I diabetic rats by increasing of P-AKT and P-ERK. According to our findings, performing daily voluntary exercise as well as taking testosterone is recommended for diabetic men with testosterone deficiency to reduce their heart complications.
